# Supplementary material for: Positive-Strand RNA Viruses Induce LTR Retrotransposon Transcription and Extrachromosomal Circular DNA Generation in Plants
Source: Int J Mol Sci. 2025 Dec 26;27(1):286. doi: 10.3390/ijms27010286 (PMC12786186; doi:10.3390/ijms27010286)
Supplement: Supplementary file 1 [file ijms-27-00286-s001.zip › File_S1.pdf]

File S1: Consensus sequences derived from LTR alignments of transcriptionally active *N. benthamiana* LTR-retrotransposons

1. Galadriel (18 elements) LTR consensus sequence

-
NtgttacggacNgcNcNaNttNNNNNccNaaNcaNNcNNaaNaNNgNNcNaNNaNgcNNNNgNa
NNagNccaNNNNNccNNgNNNggcccNNNNNNaNtcNNgaNcNNtctagaNNNNtcNNgNaNa
ttNNaNNaNNtNtagaNNNNNNNaNNNNNNNNNNNNaNNNNNNNNNNNNNNNNNNNNN
NNNNNNNNNNNNNNNNNNNNNNNNNNNNctctcNagaNctct----------
ctagaNctctNtagaNctctctagaNctctctagaN----------------------------ctctctagaactctNtaga-----
acaNtctagaaNt----------
ctcNaNaNNaNtctNNNNNNtgtatctacctagaatgtagtagaactctctagaactctccatagaNttctaggacat
gtacataNctagaaggctatagaaNtctctagaaNctNtagagctt-
gtaagcctcccNtataaataNgggatggcatttgNNatNNNNNaNNNN---------atcNNNN-
NNNNNNNagNaaaNN------------------------
tNNaagcaNNNNaagcattgtaaaNNtctcNNNNNtNtNNtNNNaNaNNagttc---------------
NtNNttNNNNNtNNtNgNttNcttcNNcNagttNNNNNaNNNNNagNcttNcttaNNNNNNNNNNa
NcNNNNggNNaNgNtgNctaNNtNNNNNNNNNNNNNNNNNNNNNNNNNgccgNacNgNtN
NNcNNNNNNaNNNtaNNNNtcNcgtNaca

2. Galadriel (15 elements) LTR consensus sequence

----------------------NtgtcacgcccctccattttctaagaggttatgccatgcggNcga---------
tgactgggcgacaNNcaggcNaNgatttggcgcacgNatggcagcatttctctagctaagtctagatccttcca---
atggaagaNtctagacccNNctagNcNNNtcttgtatattctactaNN--
------------------------tgtaNagaNN---

NNNNNNNNNttgtagaactctctagagctttgatcttagccattgattagaatagatcttagccgttgNtt--
tgggactctagcaactataaataggc-tagaggcatttggcatttgcatcc-----------
aagaaacattcaagcaatccaacacttgtaNaaagcNttctcttgtttaatacaaagcattcttgcaagcttctcttcttag---
-------cttctcttagctttgttgtggcaatatttcgggg-aaaggctgacttagcacatcgca-
ggggcgNattgtgactaaggccgcacggaagatagcta-gggagttatccggtccgtgac-----a

3. Alesia (3 elements) LTR consensus sequence

NNNNNNNNNNNNNNNNNNNNNNNN-
NNNNNNNNNNNNNNNNNNNNNNNNNNNNNNNNNNNNNNNNNNNNNNNNNNNNNNNN
NNNtatctagtagacatatctagatNttctagaatNggaaNataagaaaaatcNagatattctagagtcttggaNattta
agNaagatattagtagaaggtNatagaattgtctagattatttttgtatctagaatcatccctaNacaagtataaatagNagt
agccttaggcatttgttgtgaagcNaaaaNtcaagaaagNcttctttcNNNaacaaagttctcctNNNNNaaaNNt
NtctNNtNtNttNNaagcttccNcttcNttagttgaatcctccaatNttaNNtaacgatcttgNgctagcagaaggtctN
cgaataatacNcttcttctgctatattctttacN

4. Ivana (12 elements) LTR consensus sequence

--
Ntgtgaacaataaattaaaccaaagctagatattactagaatattctagactattctaaaatagtctagaatgagctagaat
gttctagaaggttcgagaaNattatggagttctctggaaNattctagaagatgatagaaactcttttatgtatagtagagaat
gtgagaatattctagagtagtggaata-
ttctagaacatatgagaataggtagatatgaatattgtttaggatacttatatggaatattctagaatgtcgtagaacatggag
gttgatgcctataaataggggaagacctcctcatttgtaNNatgtacaaaaNNNNNNNNNNaaagtgtgagccca
agtgtgtaccaaagtgatcccttgtgtaataaaagttgaagaattacaaaagtctctNtccatatagctaNatat-----
attaaaccttccaaac-tacctNcatttcttcatttatcaactaaacaaaatctctttcctNcttacatttaNaN-
gtcttNtaNcNttaaNttNttNtNcNNNN-

5. Ivana (5 elements) LTR consensus sequence

tgttaagattaaacttgattaacatgaggtgttctagaatcatcttgtactagctagaaaaatctttcataaaaatcaatgtatta
aactagacaattctaggaggatactagatacttctcaaataaaattcataggcatcaatctagagaattctataaacttgtat
aatcattcaagtatctagatccttccatggagtagtataaatatgtgatgttgtaactcattttgtaatcaagaaaatcaataaa
ataacaaaagccttcttcaatataaagttttatctccattccaattctctacatttcctaaactcttttcttctcattctaaatattactc
tccttctccaacaNN----
