# Supplementary material for: Positive-Strand RNA Viruses Induce LTR Retrotransposon Transcription and Extrachromosomal Circular DNA Generation in Plants
Source: Int J Mol Sci. 2025 Dec 26;27(1):286. doi: 10.3390/ijms27010286 (PMC12786186; doi:10.3390/ijms27010286)
Supplement: Supplementary file 1 [file ijms-27-00286-s001.zip › Supplementary_Figures.pdf]

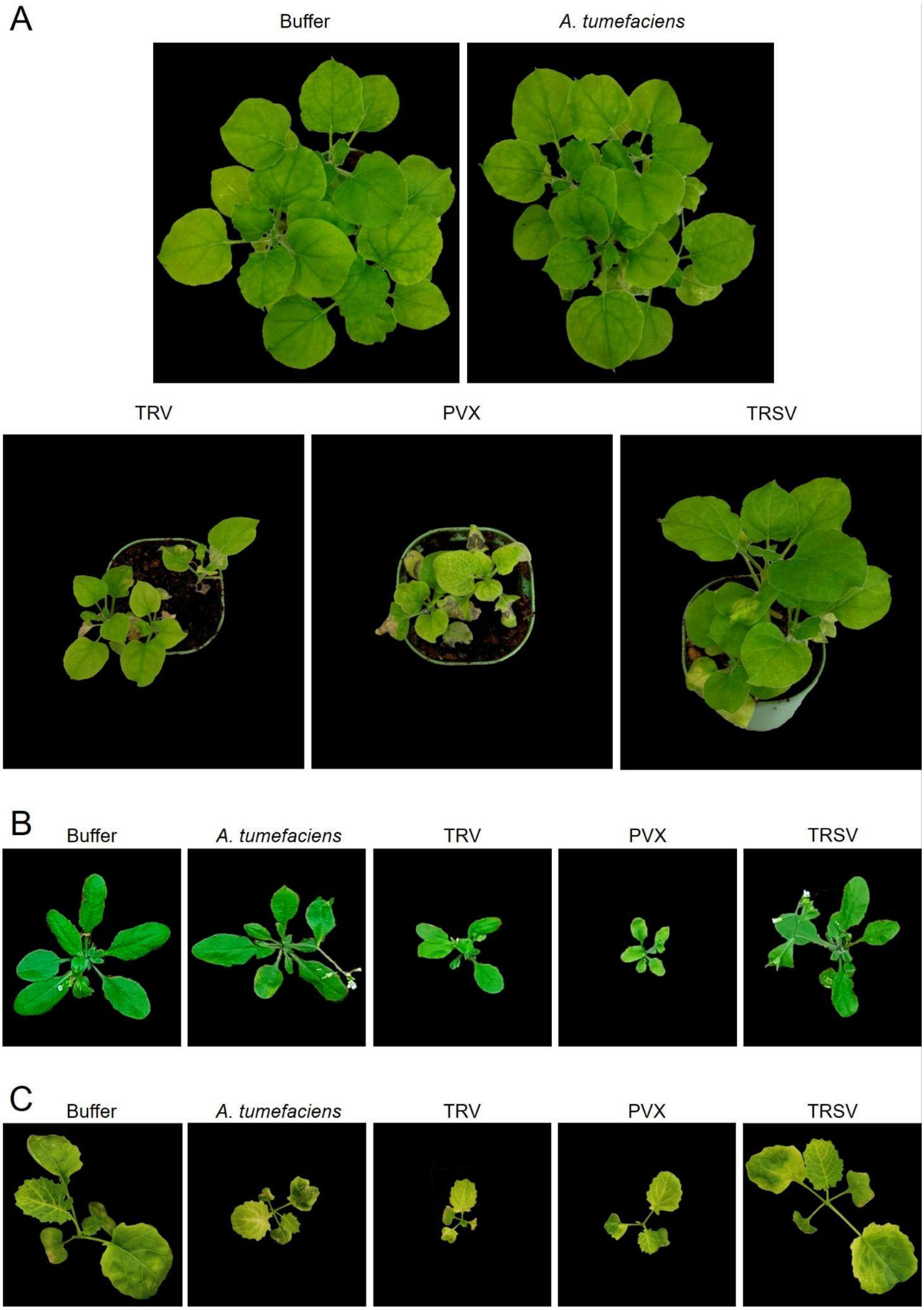

Figure S1: Phenotypic symptoms of viral infection in (A) *N. benthamiana* at 14 dpi, (B) *Arabidopsis thaliana* at 14 dpi and (C) *Brassica napus* at 7 dpi. Images for each species are presented at the same scale.

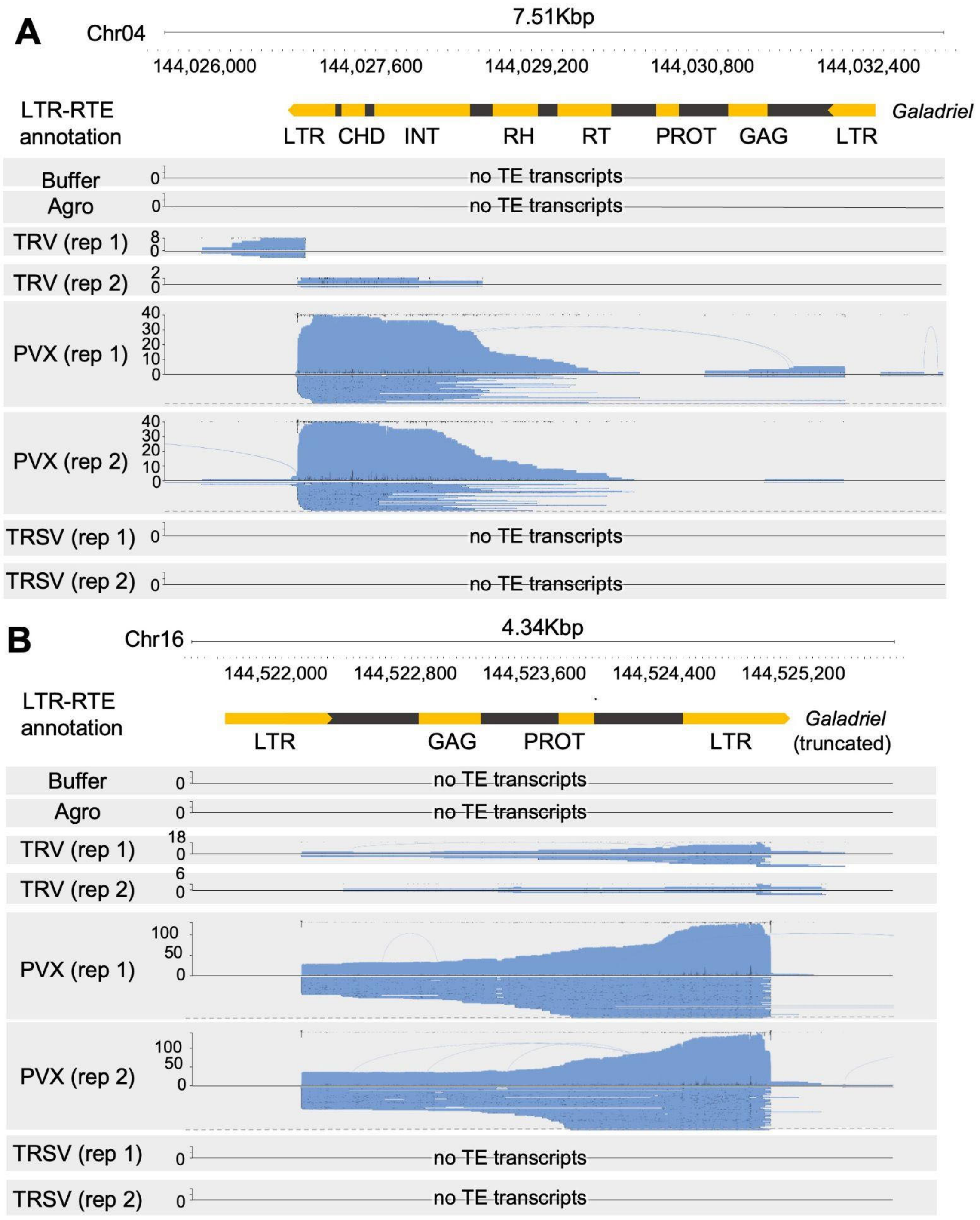

Figure S2. Genomic position, LTR-RTE structural annotation, RNA-seq coverage, and aligned reads for *Galadriel* elements from control and infected *N. benthamiana* samples. (A) Full-length *Galadriel* element on chromosome 4. (B) Truncated *Galadriel* element on chromosome 16. LTR-RTE structural annotation presented on the top, while each panel shows: RNA-seq coverage (upper blue histograms) and aligned reads (bottom section); dotted line indicates that not all aligned reads are displayed for clarity.

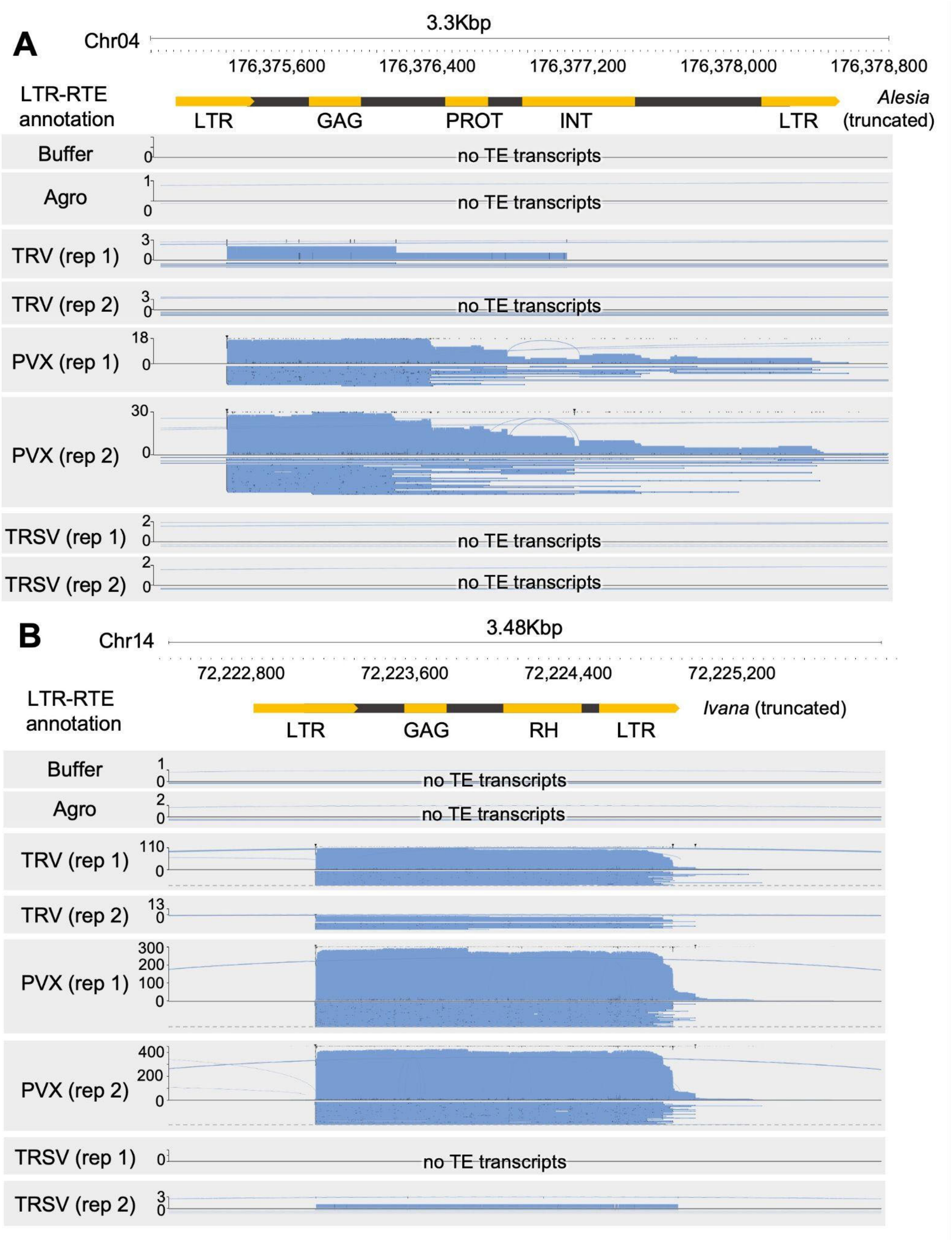

Figure S3. Genomic position, LTR-RTE structural annotation, RNA-seq coverage, and aligned reads for LTR-RTEs from control and infected *N. benthamiana* samples. (A) Truncated *Alesia* element on chromosome 4. (B) Truncated *Ivana* element on chromosome 14. LTR-RTE structural annotation presented on the top, while each panel shows: RNA-seq coverage (upper blue histograms) and aligned reads (bottom section); dotted line indicates that not all aligned reads are displayed for clarity.
